# Associations of sarcopenia, obesity, and metabolic health with the risk of urinary incontinence in U.S. adult women: a population-based cross-sectional study

**DOI:** 10.3389/fnut.2024.1459641

**Published:** 2024-10-14

**Authors:** Fei-Xue Shao, Wei-Jia Luo, Li-Qun Lou, Sheng Wan, Shi-Feng Zhao, Tian-Fan Zhou, Chen-Chen Zhou, Ying-Ying Yang, Gui-Zhu Wu, Xiao-Lin Hua

**Affiliations:** ^1^Shanghai Key Laboratory of Maternal Fetal Medicine, Shanghai Institute of Maternal-Fetal Medicine and Gynecologic Oncology, Shanghai First Maternity and Infant Hospital, School of Medicine, Tongji University, Shanghai, China; ^2^Department of Obstetrics, Shanghai First Maternity and Infant Hospital, Tongji University, Shanghai, China; ^3^Clinical Research Unit, Shanghai First Maternity and Infant Hospital, School of Medicine, Tongji University, Shanghai, China; ^4^Department of Gynecology, Shanghai First Maternity and Infant Hospital, Tongji University, Shanghai, China

**Keywords:** NHANES, DXA, sarcopenia, obesity, metabolic health, urinary incontinence

## Abstract

**Introduction:**

Urinary incontinence (UI) significantly impairs women’s quality of life. Identifying its risk factors is essential for developing effective interventions. Sarcopenia, characterized by the accelerated loss of muscle mass and function, is an emerging concern often linked to obesity and abnormal metabolic status, exacerbating various adverse health outcomes. This population-based study aimed to explore the independent and joint associations of sarcopenia, obesity, and metabolic health with UI risk, as well as to evaluate the mediating role of metabolic indicators in these associations

**Methods:**

A total of 3,557 women aged ≥20 years from the National Health and Nutrition Examination Survey were included. Sarcopenia was assessed using the appendicular lean mass index (ALMI), and obesity was defined by body mass index and waist circumference. Metabolic health was evaluated using revised criteria from the National Cholesterol Education Program-Adult Treatment Panel III. UI was identified through responses to the “Kidney Conditions-Urology” questionnaire and classified into stress UI (SUI), urgency UI (UUI), and mixed UI (MUI). Multivariable logistic regression and restricted cubic spline models were used to evaluate the associations and visualize the relationship between ALMI and UI. Mediation models were constructed to assess the mediating role of metabolic indicators.

**Results:**

We found that sarcopenia was significantly associated with an increased risk of MUI in the general population. Age-specific analysis revealed that sarcopenia is an independent risk factor for SUI in women aged ≥60, and for MUI in women aged 40–59 years. Sarcopenic obesity, particularly under central obesity criteria, further elevated the risk of UI. Notably, women with the metabolically unhealthy obese phenotype with sarcopenia were at the highest risk for both SUI and MUI. Metabolically unhealthy status, glycohemoglobin, vitamin D, and serum albumin levels were partial mediators of these associations.

**Conclusion:**

Our findings elucidated the complex interactions between sarcopenia, obesity, and metabolic health, underscoring the critical need for integrated therapeutic strategies that address both metabolic health and targeted nutritional interventions, aiming to enhance muscular health and effectively manage and prevent UI.

## 1 Introduction

Urinary incontinence (UI), defined as the involuntary leakage of urine, is a significant worldwide health concern (1, 2). It affects approximately one-third of adult women, with its prevalence increasing with age (3). This condition not only leads to social isolation and psychological distress but also imposes a substantial economic burden on society (1, 2). Given these extensive adverse effects, identifying risk factors and developing effective prevention strategies is imperative.

The etiology of UI is multifactorial, involving a complex interplay of sociodemographic factors, lifestyle choices, and chronic conditions such as aging, smoking, parity, and diabetes (3, 4). Sarcopenia, characterized by the progressive loss of skeletal muscle mass and strength, has emerged as a potential risk factor for disability, frailty, and mortality (5, 6). Despite this, the specific association between sarcopenia and UI remains poorly understood, with conflicting findings reported in the literature (7, 8).

Sarcopenia can coexist with obesity (9). Both general and central obesity are well-documented risk factors for UI, as excess body weight increases pro-inflammatory states and intra-abdominal pressure, weakening pelvic floor muscles and exacerbating UI symptoms (10, 11). The interaction between sarcopenia and obesity, often termed sarcopenic obesity, is recognized as a more accurate predictor of physical disability than either sarcopenia or obesity alone (12–14). However, the joint effect of sarcopenia and obesity on the risk of UI remains unknown.

Additionally, metabolic health plays a crucial role in the context of UI. Obesity and metabolic syndrome share multiple pathophysiological pathways. Based on metabolic characteristics such as blood pressure, glucose tolerance, and lipid profiles, individuals with obesity can be divided into two distinct phenotypes: metabolically healthy obesity (MHO) and metabolically unhealthy obesity (MUO) (15). A recent study has identified that women with the MUO phenotype faced the highest risk of UI compared to those with other phenotypes (16). Nevertheless, the contribution of sarcopenia to different obesity phenotypes in UI is underexplored, and whether metabolic health indicators mediate the link between sarcopenia or sarcopenic obesity and UI remains unclear.

To address these knowledge gaps, we conducted a population-based cross-sectional study using data from the National Health and Nutrition Examination Survey (NHANES). Our analysis aimed to elucidate the association between sarcopenia, both independently and in combination with obesity and its metabolic phenotypes, and the risk of UI among US adult women. Furthermore, we investigated the potential mediating effects of metabolic indicators, providing a deeper understanding of the relationships between sarcopenia, sarcopenic obesity, and UI.

## 2 Materials and methods

### 2.1 Study design and population

**Figure 1:**
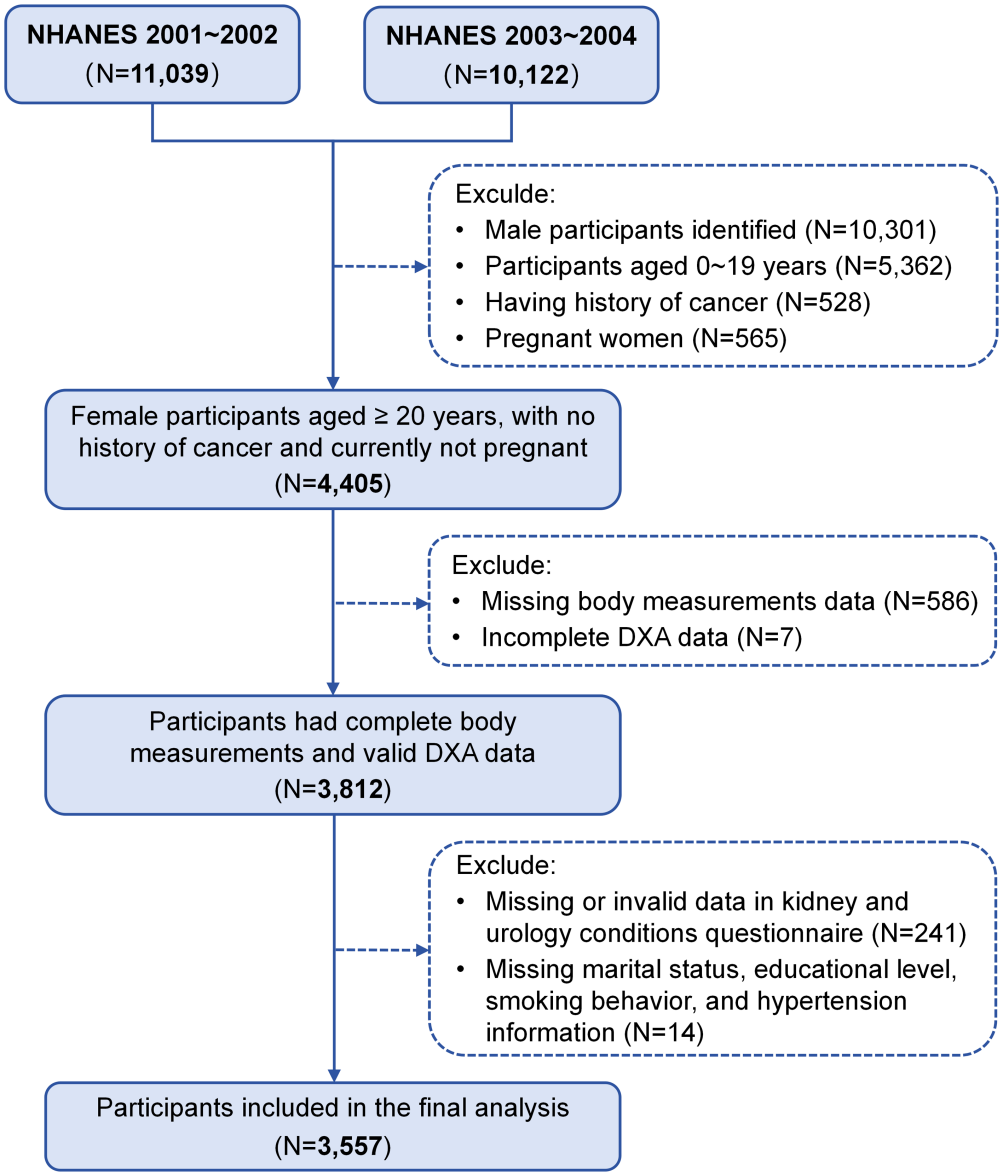
Study flow chart of participant involvement.

The NHANES is a continuous cross-sectional study conducted by the National Center for Health Statistics (NCHS) under the Centers for Disease Control and Prevention (CDC). This program aims to assess the overall health and nutritional status of the U.S. population through a nationally representative sample (17). Our study used two cycles of NHANES data from 2001 to 2004, focusing on females aged 20 years and older who underwent whole-body dual-energy x-ray absorptiometry (DXA) scans and completed the “Kidney Conditions-Urology” survey questionnaire. The exclusion criteria were as follows: male participants (*N* = 10,301), females younger than 20 years (*N* = 5,362), individuals with a history of cancer (*N* = 528), pregnant women (*N* = 565), those with incomplete data on body measurements (*N* = 586) or DXA examinations (*N* = 7), those who did not respond to the kidney conditions questionnaire (*N* = 241), and those missing covariate data (marital status, educational level, smoking behavior, and hypertension information) (*N* = 14). Consequently, a total of 3,557 participants were included in the analyses (Figure 1). This study adhered to the Strengthening the Reporting of Observational Studies in Epidemiology (STROBE) guidelines. Written informed consent was obtained from all participants, and the study protocol was approved by the NCHS Institutional Review Board (Protocol #98–12).

### 2.2 Measurements of sarcopenia, obesity, and sarcopenic obesity

Skeletal muscle mass was assessed using a whole-body DXA absorptiometry QDR-4500 scanner (Hologic, Bedford, MA, United States), known for its robustness and reproducibility (18). For safety, participants were excluded if they weighed over 136.4 kg, were taller than 192.5 cm, or had been exposed to radiographic contrast material within the past 7 days. Appendicular lean mass (ALM), defined as the sum of bone-free muscle mass in the legs and arms, was used to calculate the ALM index (ALMI) as the ratio of ALM to BMI. According to the Foundation for the National Institutes of Health (FNIH) guidelines, an ALMI of less than 0.512 was classified as sarcopenia in females (19).

Weight, height, and waist circumference (WC) were measured at mobile examination centers by trained staff using standardized procedures. BMI was calculated as weight (kg) divided by height squared (m^2^). WC was measured by placing a flexible tape around the trunk just above the top lateral edge of the ilium. Consistent with previous reports, general obesity was defined as BMI ≥30 kg/m^2^, and central obesity as WC ≥88 cm in women (20, 21). Sarcopenic obesity was defined when participants met the criteria for both sarcopenia and obesity based on these definitions.

### 2.3 Metabolic health, obesity phenotypes, and group classification

Metabolic health status was evaluated using the revised National Cholesterol Education Program-Adult Treatment Panel III (NCEP-ATP III) criteria for metabolic syndrome (22). Participants were classified as metabolically unhealthy if they had at least two of the following risk components: impaired fasting plasma glucose (FPG >100 mg/dL or diagnosed diabetes), high blood pressure (BP ≥130/85 mmHg or use of antihypertensive medication), elevated triglycerides (TG ≥150 mg/dL), and reduced high-density lipoprotein cholesterol (HDL-C < 50 mg/dL in females). Due to collinearity between BMI and waist circumference (WC), the central obesity criterion was excluded. Fasting blood samples were collected in the morning after at least 8 h of fasting and were analyzed using standardized laboratory procedures. BP was measured three times by an experienced physician to calculate the mean value.

Based on the combination of obesity and metabolic health status, four phenotypes were identified: metabolically healthy normal weight (MHNW), metabolically unhealthy normal weight (MUNW), metabolically healthy obesity (MHO), and metabolically unhealthy obesity (MUO). Women were further categorized into eight groups based on obesity phenotypes and the presence of sarcopenia: (1) MHNW without sarcopenia (MHNW/sarcopenia−); (2) MHNW with sarcopenia (MHNW/sarcopenia+); (3) MUNW without sarcopenia (MUNW/sarcopenia−); (4) MUNW with sarcopenia (MUNW/sarcopenia+); (5) MHO without sarcopenia (MHO/sarcopenia−); (6) MHO with sarcopenia (MHO/sarcopenia+); (7) MUO without sarcopenia (MUO/sarcopenia−); and (8) MUO with sarcopenia (MUO/sarcopenia+).

### 2.4 Assessment of urinary incontinence

Our primary outcome was the presence of UI, classified into three major subtypes: stress urinary incontinence (SUI), urgency urinary incontinence (UUI), and mixed urinary incontinence (MUI). These categories were identified using two specific questions from the “Kidney Conditions-Urology” questionnaire, each allowing for a binary (yes/no) response. This self-reported method for classifying UI types has been validated for its effectiveness and reliability (23, 24).

Stress UI, characterized by urine leakage during physical activities, was defined as a positive response to the question: “During the past 12 months, have you leaked or lost control of even a small amount of urine with activities like coughing, lifting, or exercise?” UUI, defined as urine leakage before reaching the toilet, was established based on a positive response to the question: “During the past 12 months, have you leaked or lost control of even a small amount of urine with an urge or pressure to urinate, and you could not get to the toilet fast enough?.” MUI was identified when positive responses were recorded for both SUI and UUI symptoms. Additionally, women who did not report either SUI or UUI were categorized as non-affected.

### 2.5 Covariates and mediators

Potential confounders and mediators were identified based on previous literature. Sociodemographic characteristics included age categories (20–39, 40–59, and ≥60 years), race/ethnicity (Mexican American, non-Hispanic white, non-Hispanic black, and others), marital status (married/living with a partner, and widowed/divorced/living alone), family poverty income ratio (PIR) (<1.3, 1.3–3.5, and >3.5), and educational level (less than high school, high school, and more than high school). Lifestyle factors included physical activity (quantified in metabolic equivalents and categorized as inactive or active) and smoking behavior (never smoked, former smoker, and current smoker). Serum vitamin D levels (nmol/L) were determined by summing the concentrations of 25-hydroxyvitamin D2 and 25-hydroxyvitamin D3. These levels were initially measured using the Dia-Sorin radioimmunoassay kit (Stillwater, MN, United States) and subsequently adjusted to align with LC–MS/MS standards for consistency. Serum albumin levels (g/dL) were measured using the Roche Cobas 6,000 analyzer (c501 module). Chronic health conditions were defined as follows: diabetes (FPG ≥126 mg/dL, HbA1c ≥6.5%, diagnosis by a physician, or use of hypoglycemic medication), pre-diabetes (FPG 100–125 mg/dL or HbA1c 5.7–6.4%) (25), and hypertension (mean systolic/diastolic BP ≥140/90 mmHg or current use of BP-lowering medication). Reproductive factors included parity (number of live births, categorized as 1, 2, 3, and ≥4), and use of female hormones (yes/no).

### 2.6 Statistical analyses

All data analyses were conducted using R software, version 4.2.2 (R Foundation for Statistical Computing, Vienna, Austria; http://www.r-project.org). A two-tailed *p*-value <0.05 was considered statistically significant. Given the NHANES survey’s complex, multistage, stratified probability sampling method, we used the mobile examination center exam sample weights for analysis, following the analytical and reporting guidelines. To address potential biases due to non-random missing DXA data, multiple imputations were conducted by NCHS, resulting in five complete DXA datasets for each participant^1^.

In the descriptive analysis, continuous variables were presented as means with standard deviations (SD) and analyzed using Student’s *t*-test, while categorical data were expressed as counts with weighted percentages (%) and evaluated using Pearson’s *χ*^2^ test (R package “TableOne”). Multivariable logistic regression models were initially employed to estimate weighted odds ratios (OR) with 95% confidence intervals (CI) for the independent effect of sarcopenia on UI. These models were subsequently used to evaluate the joint associations of sarcopenia and obesity, including its phenotypes, on UI subtypes. Potential covariates were selected based on existing literature and data availability. We confirmed no collinearity between the exposures and covariates for UI risk, using a variance inflation factor threshold of less than 10. For missing categorical covariates (family PIR and physical activity), the missing indicator approach was applied. Two models were developed through different stages: Model 1 accounted for sociodemographic factors (age categories, race/ethnicity, marital status, educational level, and family PIR). Model 2 was further adjusted for smoking behavior, physical activity, diabetes, hypertension, parity, and female hormone use. To test the heterogeneity of the association between sarcopenia and UI, subgroup analyses were performed, stratified by smoking behavior, physical activity, hypertension, diabetes, and parity. Survey design and weighting variables were considered in all models (R package “survey”), and results across the five imputed datasets were consolidated according to Rubin’s rules (R package “mitools”) (26, 27).

To account for the exposure-response relationship between continuous ALMI and UI subtypes, restricted cubic splines (RCS) were performed across the five imputed datasets, adjusted for the same confounders as in regression model 2 (R package “rms”). Each RCS curve was constructed with four knots positioned at the 5th, 35th, 65th, and 95th percentiles of ALMI, with the reference ALMI set at OR = 1. The five curves resulting from the RCS models were combined and displayed in a single figure to present a unified view.

In addition, mediation analyses were conducted to assess the mediating role of metabolic indicators (metabolic unhealthy status, HbA1c, vitamin D, and albumin levels) on the relationship between sarcopenia, sarcopenic obesity, and UI (R package “Mediation”). These analyses were performed on each of the five imputed datasets with 1,000 bootstrap simulations. To minimize confounding bias, all mediation models were adjusted for the covariates in Model 2. The direct effect measured the impact of sarcopenia or sarcopenic obesity on UI without the mediator, while the indirect effect measured the impact through the mediators. The proportion of mediation was calculated as (indirect effect/total effect) × 100% to quantify the mediating effect size.

## 3 Results

### 3.1 Baseline characteristics

Baseline characteristics of women categorized by three UI subtypes are shown in Supplementary Table S1. Among the 3,557 eligible women, 39.6% had SUI, 23.2% had UUI, and 15.1% had MUI. Women reporting SUI were predominantly non-Hispanic white, while those with UUI or MUI were more likely to have lower educational levels and lower family PIR ratios (<1.3). Across all UI subtypes, women with UI were significantly older, more likely to be married or living with a partner, and exhibited higher incidences of smoking (both former and current), obesity, physical inactivity, pre-diabetes or diabetes, higher parity, and use of female hormones compared to those without UI (all *p*-values <0.05).

### 3.2 Association of sarcopenia on UI risk

Supplementary Figure S1 displays the distribution of DXA parameters (lean muscle mass in the left arm, right arm, left leg, right leg, and appendicular regions) across five imputed datasets. The density curves for each parameter exhibit significant overlap, indicating a consistent distribution pattern across these datasets. The ALMI was derived from these DXA parameters, with sarcopenia defined as ALMI <0.512.

Table 1 details the relationship between sarcopenia and three subtypes of UI. In the entire cohort, sarcopenia was significantly associated with an increased risk of MUI (OR = 1.541, 95% CI: 1.082–2.195), whereas no significant associations were observed with SUI or UUI. Age-specific analyses revealed distinct patterns: among women aged ≥60 years, sarcopenia was significantly correlated with an elevated risk of SUI (OR = 1.576, 95% CI: 1.099–2.259), while among women aged 40–59 years, it was associated with a higher risk of MUI (OR = 2.022, 95% CI: 1.122–3.642). These associations were not evident in women aged 20–39 years, and no significant correlation was observed between sarcopenia and UUI in any age group. RCS models were used to visualize the relationship between continuous ALMI and UI subtypes (Supplementary Figure S2). For MUI, a significant negative linear relationship was observed across the entire range of ALMI values. When stratified by age groups, the most pronounced associations were found in women aged ≥60 years for SUI and in the 40–59 age group for MUI, consistent with the primary analysis.

**Table 1:** Association of sarcopenia with UI subtypes stratified by age groups (combined 5 imputed datasets).

	All women		20 ~ 39 years		40 ~ 59 years		≥60 years	
	Model 1^a^ OR (95% CI)	Model 2^b^ OR (95% CI)	Model 1^c^ OR (95% CI)	Model 2^d^ OR (95% CI)	Model 1^c^ OR (95% CI)	Model 2^d^ OR (95% CI)	Model 1^c^ OR (95% CI)	Model 2^d^ OR (95% CI)
Stress urinary incontinence								
Non-sarcopenia	1.00 (Reference)	1.00 (Reference)	1.00 (Reference)	1.00 (Reference)	1.00 (Reference)	1.00 (Reference)	1.00 (Reference)	1.00 (Reference)
Sarcopenia	**1.312 (1.038, 1.657)** *	1.253 (0.982, 1.599)	1.089 (0.531, 2.232)	1.031 (0.474, 2.246)	1.110 (0.663, 1.859)	1.084 (0.627, 1.873)	**1.576 (1.152, 2.158)** *	**1.576 (1.099, 2.259)** *
Urgency urinary incontinence								
Non-sarcopenia	1.00 (Reference)	1.00 (Reference)	1.00 (Reference)	1.00 (Reference)	1.00 (Reference)	1.00 (Reference)	1.00 (Reference)	1.00 (Reference)
Sarcopenia	1.221 (0.887, 1.682)	1.170 (0.822, 1.666)	1.320 (0.562, 3.102)	1.363 (0.556, 3.345)	1.564 (0.935, 2.619)	1.477 (0.866, 2.518)	0.956 (0.625, 1.462)	0.898 (0.563, 1.431)
Mixed urinary incontinence								
Non-sarcopenia	1.00 (Reference)	1.00 (Reference)	1.00 (Reference)	1.00 (Reference)	1.00 (Reference)	1.00 (Reference)	1.00 (Reference)	1.00 (Reference)
Sarcopenia	**1.617 (1.174, 2.226)** *	**1.541 (1.082, 2.195)** *	1.312 (0.450, 3.823)	1.246 (0.411, 3.781)	**2.183 (1.220, 3.906)** *	**2.022 (1.122, 3.642)** *	1.291 (0.814, 2.047)	1.280 (0.777, 2.108)

UI, urinary incontinence; OR, odds ratio; CI, confidence interval.

a Adjusted for age categories, race/ethnicity, marital status, educational level and family PIR.

b Adjusted for covariables in subscript “a” plus smoking behavior, physical activities, diabetes, hypertension, parity, and female hormone use.

c Adjusted for race/ethnicity, marital status, educational level and family PIR.

d Adjusted for covariables in subscript “c” plus smoking behavior, physical activities, diabetes, hypertension, parity, and female hormone use. *Statistically significant and presented in bold text.

Subgroup analyses were conducted to assess the heterogeneity of these associations. As shown in Supplementary Table S2, sarcopenia was significantly associated with MUI among former smokers, individuals without hypertension, inactive individuals, those with pre-diabetes or diabetes, and women with three childbirths. Significant associations with SUI were also observed among inactive individuals, those with pre-diabetes, and women with two childbirths. No significant association was found between sarcopenia and UUI in any subgroup.

### 3.3 Joint association of sarcopenia, obesity, and metabolic health on UI risk

The joint analysis of sarcopenia and obesity on the risk of UI is presented in Table 2. Women were categorized into four groups: control, sarcopenia alone, obesity alone, and sarcopenic obesity. Compared to the control group, women in the obesity alone and sarcopenic obesity groups demonstrated elevated risk for SUI and MUI, with the highest risks in the sarcopenic obesity group, particularly under the central obesity definition (OR = 2.345, 95% CI 1.773–3.101 for SUI; OR = 2.771, 95% CI 1.729–4.441 for MUI). For UUI, the obesity alone group consistently showed significant positive associations (OR = 1.540, 95% CI 1.286–1.844 in general obesity; OR = 1.671, 95% CI 1.307–2.138 in central obesity), while sarcopenic obesity was significant only with central obesity (OR = 1.755, 95% CI 1.114–2.765).

**Table 2:** Joint association of sarcopenia and obesity with the risk of UI subtypes in all women (combined 5 imputed datasets).

	Obesity defined by BMI		Obesity defined by WC	
	Model 1^a^ OR (95% CI)	Model 2^b^ OR (95% CI)	Model 1^c^ OR (95% CI)	Model 2^d^ OR (95% CI)
Stress urinary incontinence				
Control	1.00 (Reference)	1.00 (Reference)	1.00 (Reference)	1.00 (Reference)
Sarcopenia alone	1.077 (0.799, 1.453)	1.106 (0.816, 1.498)	0.830 (0.409, 1.682)	0.775 (0.377, 1.593)
Obesity alone	**1.799 (1.479, 2.190)** *	**1.755 (1.432, 2.151)** *	**2.043 (1.707, 2.445)** *	**1.971 (1.648, 2.358)** *
Sarcopenic obesity	**1.979 (1.429, 2.740)** *	**1.915 (1.348, 2.719)** *	**2.345 (1.773, 3.101)** *	**2.307 (1.745, 3,050)** *
Urgency urinary incontinence				
Control	1.00 (Reference)	1.00 (Reference)	1.00 (Reference)	1.00 (Reference)
Sarcopenia alone	1.216 (0.822, 1.798)	1.232 (0.814, 1.865)	1.579 (0.896, 2.780)	1.588 (0.916, 2.753)
Obesity alone	**1.540 (1.286, 1.844)** *	**1.523 (1.250, 1.855)** *	**1.671 (1.307, 2.138)** *	**1.666 (1.289, 2.153)** *
Sarcopenic obesity	1.533 (0.946, 2.486)	1.481 (0.861, 2.548)	**1.755 (1.114, 2.765)** *	**1.729 (1.074, 2.783)** *
Mixed urinary incontinence				
Control	1.00 (Reference)	1.00 (Reference)	1.00 (Reference)	1.00 (Reference)
Sarcopenia alone	1.571 (0.993, 2.486)	**1.656 (1.044, 2.628)** *	**1.903 (1.120, 3.234)** *	**1.894 (1.112, 3.226)** *
Obesity alone	**1.775 (1.433, 2.198)** *	**1.721 (1.350, 2.193)** *	**2.026 (1.511, 2.715)** *	**1.989 (1.450, 2.728)** *
Sarcopenic obesity	**2.246 (1.381, 3.652)** *	**2.106 (1.197, 3.706)** *	**2.771 (1.729, 4.441)** *	**2.715 (1.652, 4.462)** *

UI, urinary incontinence; BMI, body mass index; WC, waist circumference; OR, odds ratio; CI, confidence interval.

a Adjusted for age categories, race/ethnicity, marital status, educational level and family PIR.

b Adjusted for covariables in subscript “a” plus smoking behavior, physical activities, diabetes, hypertension, parity, and female hormone use.

c Adjusted for race/ethnicity, marital status, educational level and family PIR.

d Adjusted for covariables in subscript “c” plus smoking behavior, physical activities, diabetes, hypertension, parity, and female hormone use. *Statistically significant and presented in bold text.

**Figure 2:**
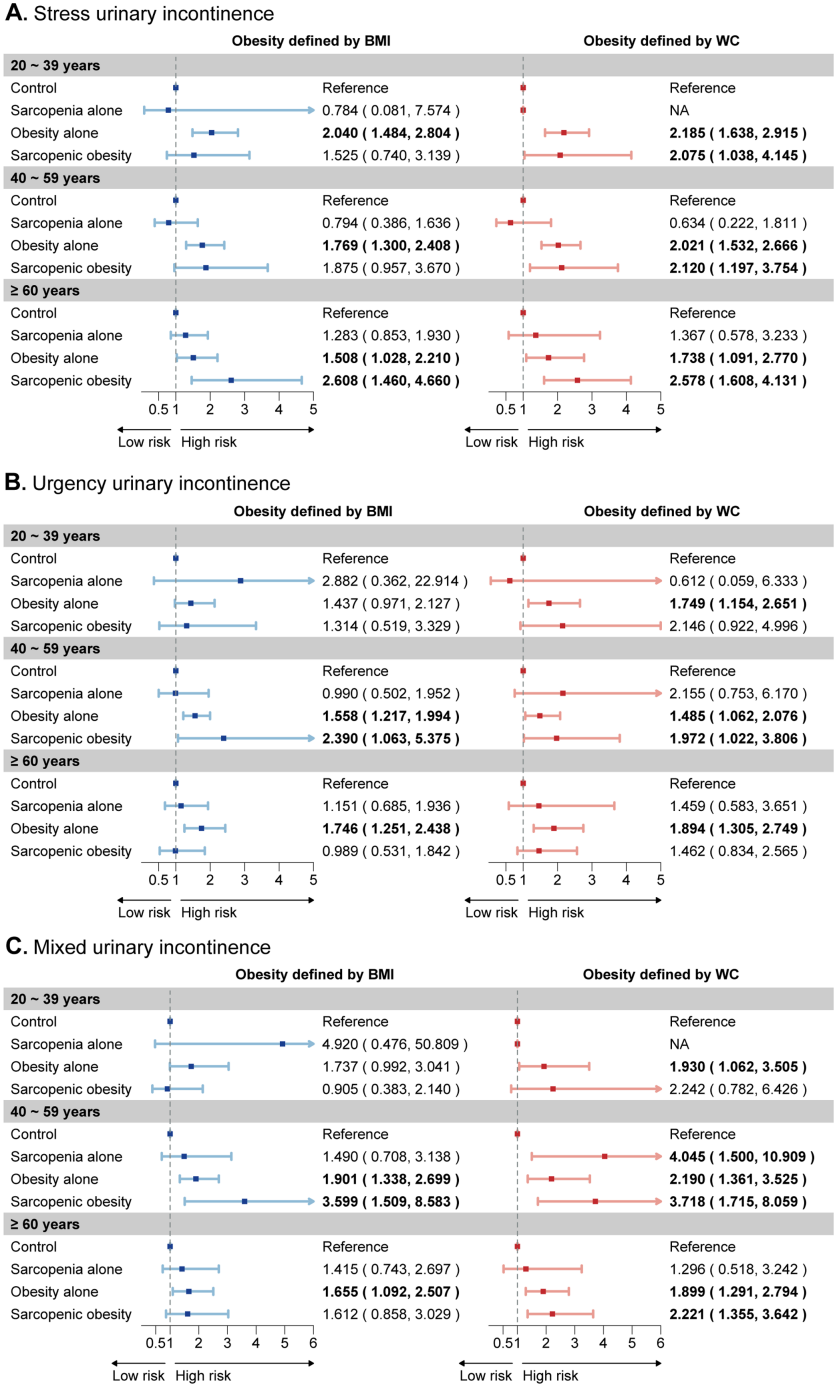
Age-specific subgroup analysis of the joint effects of sarcopenia and obesity on UI risk. The forest plot illustrates the estimated effects of sarcopenia, obesity, and their joint effect on the risk of **(A)** stress urinary incontinence, **(B)** urgency urinary incontinence, and **(C)** mixed urinary incontinence across three age categories. These categories are depicted on the y-axis for each age group. Weighted odds ratios (OR) and 95% confidence intervals (CI) are presented by dots and horizontal lines, respectively. Statistically significant relationships are highlighted in bold text for clarity. UI, urinary incontinence.

Age-specific analysis suggested the impact of sarcopenic obesity on UI risk varies across age groups and differs depending on the obesity definitions used (Figure 2). Using the general obesity definition, sarcopenic obesity was significantly associated with SUI in women aged ≥60 years, and with UUI and MUI in those aged 40–59 years. Under the central obesity definition, sarcopenic obesity showed a significant correlation with SUI across all age groups. For UUI, it was significant only in those aged 40–59 years, while for MUI, it was significant among middle-aged and older women.

**Figure 3:**
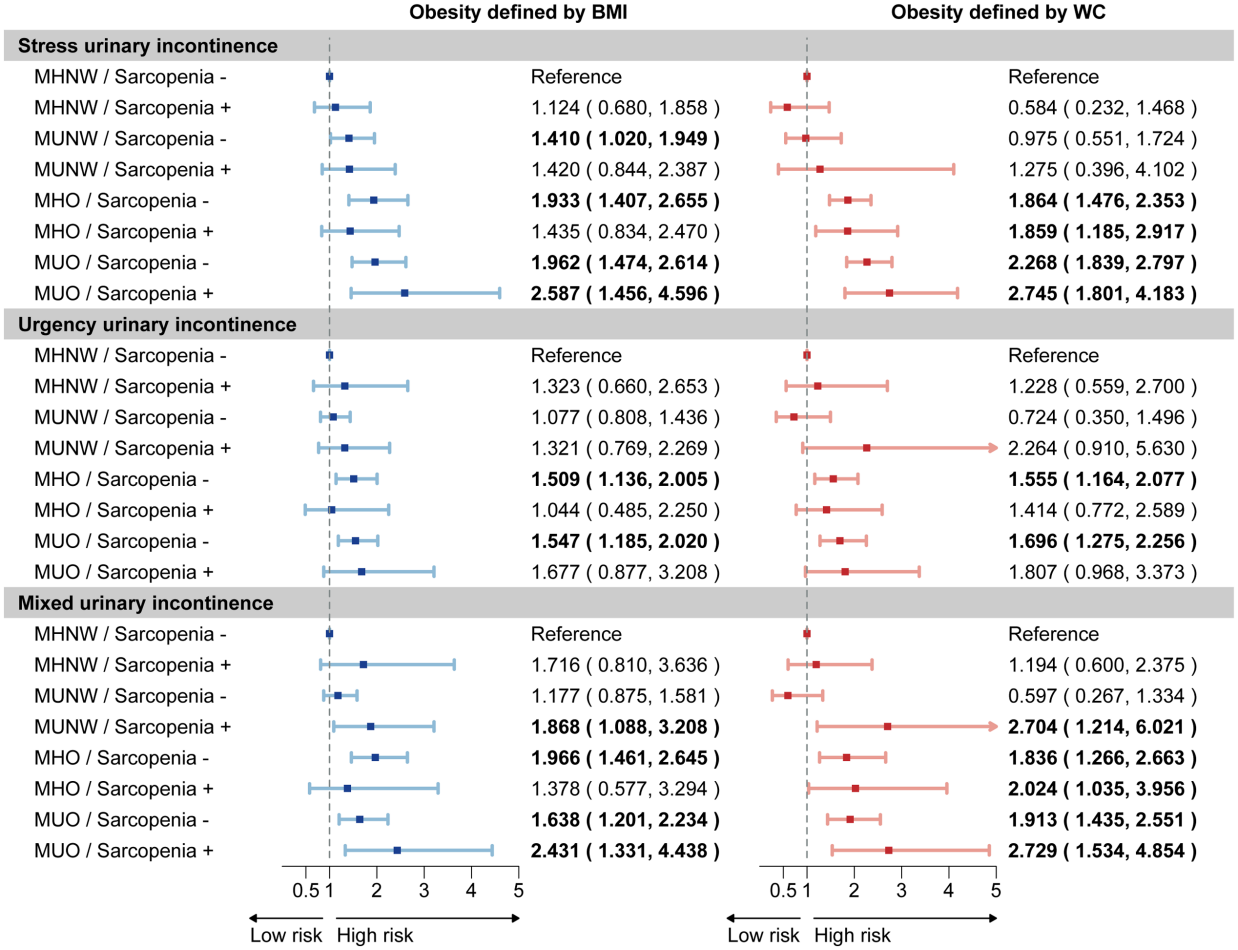
Joint association of sarcopenia and obesity phenotypes on UI risk. The forest plot illustrates the estimated effects of sarcopenia and obesity phenotypes on the risk of stress urinary incontinence, urgency urinary incontinence, and mixed urinary incontinence. Weighted odds ratios (OR) and 95% confidence intervals (CI) are presented by dots and horizontal lines, respectively. Statistically significant relationships are highlighted in bold text for clarity. The analysis was conducted using a sample size of 3,380 women. UI, urinary incontinence; MHNW, metabolic healthy normal weight; MUNW, metabolic unhealthy normal weight; MHO, metabolic healthy obesity; MUO, metabolic unhealthy obesity.

We further investigated the joint association between sarcopenia and obesity phenotypes on UI risk, dividing participants into eight groups based on the presence of sarcopenia, obesity, and metabolic status, with the MHNW/sarcopenia (−) group serving as the reference (Figure 3). Our joint analysis revealed that sarcopenia increased the risk ratios for MUI in the MUO, MHO, and MUNW phenotypes, with the strongest association observed in the MUO (+)/sarcopenia (+) group, particularly under central obesity criteria. Moreover, sarcopenia combined with the MUO phenotype showed the highest risk for SUI, while no similar association was found for UUI.

### 3.4 Mediation analyses

**Figure 4:**
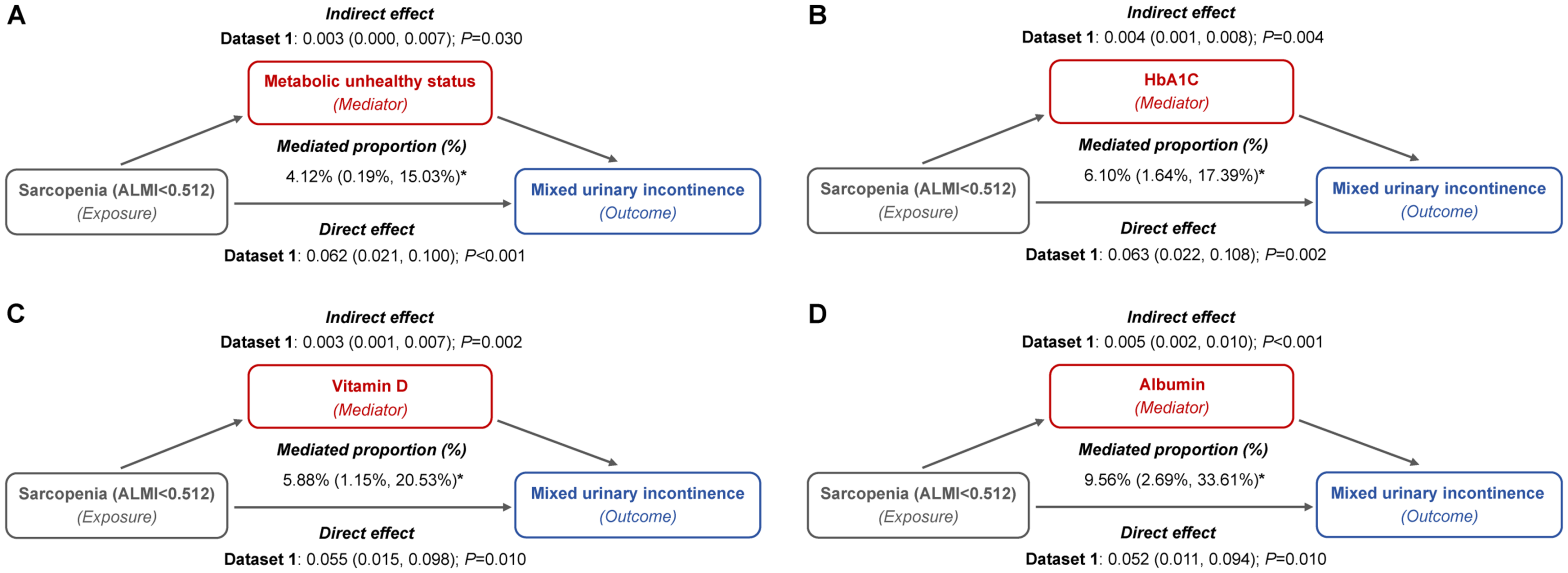
Mediation path models of metabolic indicators in the relationship between sarcopenia and MUI (imputed dataset 1). **(A)** Metabolically unhealthy status: this model included a sample size of 3,327 women and was adjusted for age categories, race/ethnicity, marital status, educational level, family PIR, smoking behavior, physical activity, parity, and female hormone use. **(B)** HbA1c: this model included a sample size of 3,389 women and was adjusted for age categories, race/ethnicity, marital status, educational level, family PIR, smoking behavior, physical activity, hypertension, parity, and female hormone use. **(C)** Vitamin D: this model included a sample size of 3,331 women and was adjusted for age categories, race/ethnicity, marital status, educational level, family PIR, smoking behavior, physical activity, hypertension, diabetes, parity, and female hormone use. **(D)** Albumin: this model included a sample size of 3,325 women and was adjusted for age categories, race/ethnicity, marital status, educational level, family PIR, smoking behavior, physical activity, hypertension, diabetes, parity, and female hormone use.

We investigated the mediating role of metabolic indicators in the relationship between sarcopenia and MUI across five imputed datasets. Figure 4 depicts the mediation path models for the imputed dataset1, while results from the other four datasets are presented in Supplementary Table S3. Our findings consistently demonstrated a significant direct effect of sarcopenia on MUI. Furthermore, we identified a positive indirect effect through metabolically unhealthy status, HbA1c, serum vitamin D, and albumin levels, with maximum mediation effects of 4.36, 6.58, 6.68, and 10.33%, respectively (Supplementary Table S3).

**Figure 5:**
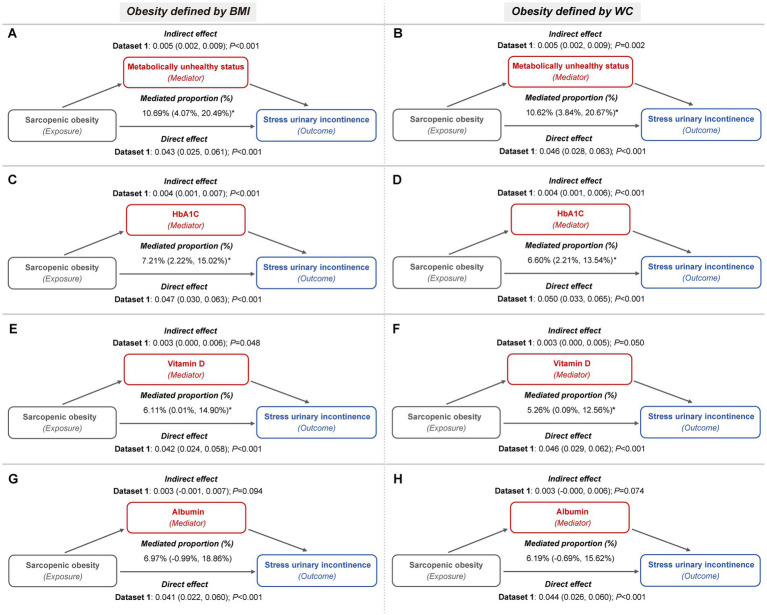
Metabolic indicators mediation path models of the relationship of sarcopenic obesity on SUI (imputed dataset 1). **(A,B)** The mediating role of metabolically unhealthy status, with obesity defined by BMI **(A)** and WC **(B)**, respectively. Both mediation models included a sample size of 3,327 women and were adjusted for age categories, race/ethnicity, marital status, educational level, family PIR, smoking behavior, physical activity, parity, and female hormone use. **(C,D)** The mediating role of HbA1c, with obesity defined by BMI **(C)** and WC **(D)**, respectively. Both mediation models included a sample size of 3,389 women and were adjusted for age categories, race/ethnicity, marital status, educational level, family PIR, smoking behavior, physical activity, hypertension, parity, and female hormone use. **(E,F)** The mediating role of vitamin D, with obesity defined by BMI **(E)** and WC **(F)**, respectively. Both mediation models included a sample size of 3,331 women and were adjusted for age categories, race/ethnicity, marital status, educational level, family PIR, smoking behavior, physical activity, hypertension, diabetes, parity, and female hormone use. **(G,H)** The mediating role of albumin, with obesity defined by BMI **(G)** and WC **(H)**, respectively. Both mediation models included a sample size of 3,325 women and were adjusted for age categories, race/ethnicity, marital status, educational level, family PIR, smoking behavior, physical activity, hypertension, diabetes, parity, and female hormone use.

**Figure 6:**
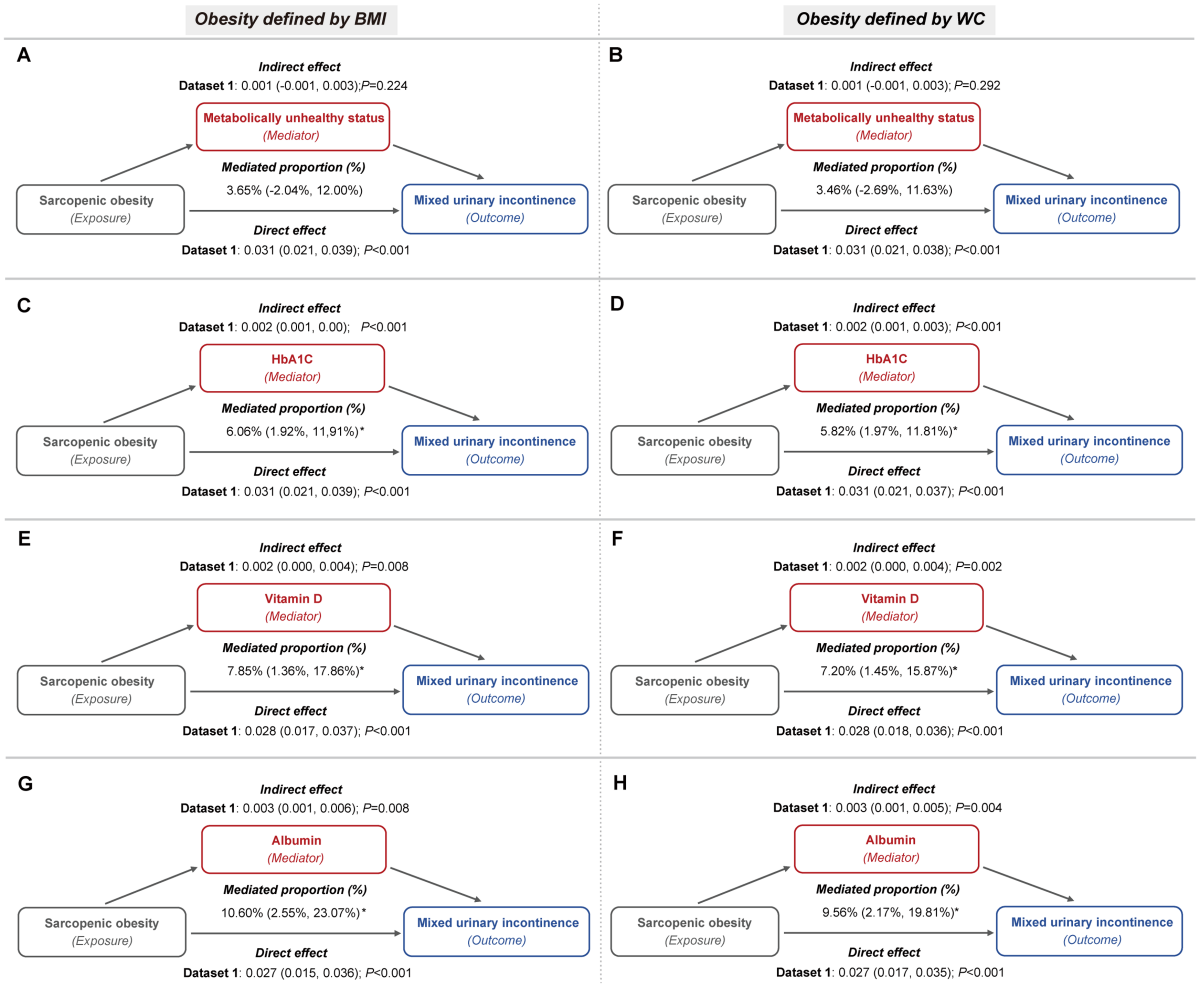
Metabolic indicators mediation path models of the relationship of sarcopenic obesity on MUI (imputed dataset 1). **(A,B)** The mediating role of metabolically unhealthy status, with obesity defined by BMI **(A)** and WC **(B)**, respectively. Both mediation models included a sample size of 3,327 women and were adjusted for age categories, race/ethnicity, marital status, educational level, family PIR, smoking behavior, physical activity, parity, and female hormone use. **(C,D)** The mediating role of HbA1c, with obesity defined by BMI **(C)** and WC **(D)**, respectively. Both mediation models included a sample size of 3,389 women and were adjusted for age categories, race/ethnicity, marital status, educational level, family PIR, smoking behavior, physical activity, hypertension, parity, and female hormone use. **(E,F)** The mediating role of vitamin D, with obesity defined by BMI **(E)** and WC **(F)**, respectively. Both mediation models included a sample size of 3,331 women and were adjusted for age categories, race/ethnicity, marital status, educational level, family PIR, smoking behavior, physical activity, hypertension, diabetes, parity, and female hormone use. **(G,H)** The mediating role of albumin, with obesity defined by BMI **(G)** and WC **(H)**, respectively. Both mediation models included a sample size of 3,325 women and were adjusted for age categories, race/ethnicity, marital status, educational level, family PIR, smoking behavior, physical activity, hypertension, diabetes, parity, and female hormone use.

These mediators also partially accounted for the association between sarcopenic obesity and the risk of both SUI and MUI. For SUI, metabolically unhealthy status mediated up to 11.44 and 11.35% of the association, while HbA1c contributed to 7.65 and 7.01%, and vitamin D to 6.42 and 5.51%, depending on whether general or central obesity criteria were applied (Figure 5; Supplementary Table S4). In the case of MUI, HbA1c mediated up to 6.34 and 6.08%, serum vitamin D mediated up to 8.33 and 7.61%, and serum albumin mediated up to 10.99 and 9.92%, under the general and central obesity definitions, respectively. Conversely, metabolically unhealthy status did not exhibit a significant mediation effect in this association (Figure 6; Supplementary Table S5).

## 4 Discussion

In this study, we found a significant association between sarcopenia and MUI among the general participants. After stratifying by age groups, sarcopenia emerged as an independent risk factor for SUI in women aged ≥60 years, and for MUI in women aged 40–59 years. A joint analysis of sarcopenia and obesity indicated that sarcopenic obesity posed an increased risk of UI, particularly under central obesity criteria. Further stratification based on sarcopenia and obesity phenotypes revealed that women in the MUO phenotype with sarcopenia faced the highest risk for both SUI and MUI. Additionally, metabolically unhealthy status, HbA1c, vitamin D and albumin levels partially mediated the association between sarcopenia and MUI, as well as between sarcopenic obesity and SUI or MUI.

Previous studies on the relationship between sarcopenia and UI have yielded inconsistent results. Park et al. (28) found no significant correlation between muscle loss and UI in older Korean women, while Erdogan et al. (8) conducted a European cohort and identified sarcopenia as an independent predictor of SUI and UUI. These discrepancies may arise from variations in study populations, definitions of sarcopenia, and consideration of confounding factors such as diabetes, smoking, and reproductive history (7, 28). Our study adopted the FNIH’s definition of sarcopenia, which is considered biologically plausible and more appropriate for U.S. women (19). We carefully selected confounders based on data availability and prior literature to reduce bias (1, 2). Our findings revealed that sarcopenia was associated with a 1.6-fold increased risk of SUI in women aged ≥60 years and a 2.2-fold increased risk of MUI in those aged 40–59 years, with no significant link to UUI. This differential impact may reflect the distinct pathophysiological mechanisms underlying SUI and UUI, with SUI primarily related to urethral closure deficiency and UUI often associated with impaired sensory pathways (29). Given that sarcopenia predominantly affects skeletal muscles, including those crucial for pelvic and abdominal support, impairments in urethral pressure generation may increase the risk of SUI, especially in older women (29). Moreover, sarcopenia demonstrated a more pronounced association with MUI compared to SUI. This observation can be explained by the heterogeneous nature of MUI, which encompasses various bladder control issues, and the interaction between causative factors for SUI and UUI contributes to the complexity and severity of MUI (30).

Obesity is widely recognized as an independent risk factor for all UI subtypes (10, 31). Recent studies have identified a bidirectional relationship between sarcopenia and obesity. Sarcopenia can reduce resting metabolic rates and total energy expenditure, promoting fat accumulation (9). Conversely, intramuscular lipid accumulation may induce lipotoxicity and oxidative damage, leading to the degradation of vital muscle proteins and compromised muscle fiber contractility (14, 32). The existing literature has suggested that the coexistence of sarcopenia and obesity, known as sarcopenic obesity, increases the risk of disability, frailty, and mortality more than either condition alone (9, 13). However, evidence of the association between sarcopenic obesity and UI has been lacking. Our study conducted a joint analysis of sarcopenia and obesity, indicating that women with sarcopenic obesity faced the highest risk of all three subtypes of UI. This connection was even stronger when obesity was defined using WC. WC is a valuable indicator of central obesity as it specifically reflects abdominal fat, particularly visceral fat (33). The accumulation of central adiposity may exacerbate abdominal pressure and induce chronic inflammation in the pelvic floor muscles, significantly increasing their susceptibility to dysfunction and impairing urinary control (14, 34). Additionally, age-specific analyses showed that these associations were particularly robust in middle-aged and older women. This may be due to aging muscles being more prone to atrophy and ectopic fat deposition, impairing the mechanical properties of abdominal and pelvic support structures and causing neuron loss and denervation of pelvic muscle fibers (35, 36).

Obesity is also recognized as a heterogeneous condition with varying metabolic statuses, which can be categorized into MHO and MUO phenotypes based on the presence of metabolic health abnormalities (37, 38). Individuals with the MUO phenotype are associated with a heightened inflammatory state and increased oxidative stress, leading to higher rates of frailty, morbidity, and mortality (39). Previous studies have confirmed that metabolically unhealthy states, such as dyslipidemia and diabetes, contribute to the onset of UI (40–44). Fwu et al. (16) further indicated that the MUO phenotype had the strongest association with UI compared to those in the MHNW status. Given that a combination of sarcopenia and obesity is more likely associated with metabolic disorders, including type 2 diabetes and cardiovascular diseases (45, 46), we made the first attempt to perform a combined analysis to determine the extent to which sarcopenia affects the risk of UI across various metabolic obesity phenotypes. Our findings revealed that for SUI and MUI, women in the MUO phenotype with sarcopenia exhibited the greatest risk compared to other groups. Furthermore, sarcopenia also exacerbated the risk of MUI in MUNW, MHO, and MUO phenotypes. The heightened risk observed emphasized a synergistic effect, where the coexistence of metabolic unhealthiness and muscle degradation significantly increased vulnerability to UI. Routine screening for sarcopenia in obese patients and those with metabolic abnormalities is recommended to identify individuals at greater risk for UI (47).

Moreover, these findings provide valuable insights for the development of targeted intervention strategies. Over the past few decades, numerous studies have consistently demonstrated that weight loss interventions, including exercise, dietary control, and surgery, can significantly reduce the prevalence of UI (48, 49). However, it is crucial to acknowledge that conventional weight loss methods often result in the loss of both muscle mass and fat, which may be detrimental to patients with sarcopenia (50). Current evidence indicates that lifestyle interventions, particularly aerobic and resistance training, are effective in preserving muscle mass while reducing body fat, thereby improving outcomes in individuals with sarcopenic obesity (51, 52). Additionally, nutritional strategies, such as low-glycemic-index diets and adequate protein intake, have been shown to maintain muscle mass during fat loss. Supplementation with compounds such as vitamin D, creatine, leucine, and branched-chain amino acids has also demonstrated potential therapeutic benefits for patients with sarcopenic obesity (53–56). Our mediation analyses identified several key metabolic indicators, including metabolically unhealthy status, HbA1c, vitamin D levels, and albumin levels, as significant mediators in the relationship between sarcopenia, sarcopenic obesity, and the incidence of SUI and MUI. These findings suggested that improving metabolic health, adopting a high-protein diet, and ensuring adequate vitamin D supplementation may be essential in reducing the risk of UI in individuals with sarcopenia or sarcopenic obesity. Future research should focus on evaluating integrated therapeutic strategies that simultaneously address muscle preservation and metabolic health, with the ultimate goal of mitigating UI symptoms and improving clinical outcomes.

Our study had several notable strengths. First, it was the first to evaluate the independent and joint associations of sarcopenia, obesity, and metabolic health status with UI. We used data from a national, population-based database, known for its rigorous random sampling methodology, ensuring high representativeness of our findings. Second, we identified the mediating roles of metabolically unhealthy status and HbA1c in the associations between sarcopenia or sarcopenic obesity and UI. Third, we employed DXA, a gold-standard method for accurately measuring lean muscle mass and diagnosing sarcopenia. We also integrated two distinct definitions of obesity to elucidate the specific impacts of central and general obesity in the context of sarcopenia on UI. Additionally, detailed subgroup analyses were also conducted to test heterogeneity and enhance our understanding of high-risk populations.

There were also limitations that should be mentioned. First, the definition of sarcopenia in our study focused exclusively on muscle mass loss, neglecting muscle dysfunction due to the absence of this information in the NHANES 2001–2004 dataset. Second, the non-random nature of missing data in the DXA parameters posed a limitation. To mitigate this bias, we used multiple imputed datasets from the NHCS, which showed consistent distribution patterns across five datasets. Third, our study was a cross-sectional analysis, which precludes establishing a causal relationship between sarcopenia, obesity, or their metabolic phenotypes and UI. Despite efforts to adjust for multiple confounders, residual confounding from unmeasured or unknown factors may still be present. Our findings need to be confirmed through longitudinal and prospective studies in the future.

## 5 Conclusion

In summary, our findings highlighted the complex interplay between sarcopenia, obesity, and metabolic health on UI subtypes, emphasizing the importance of early screening and diagnosis of sarcopenia, particularly in women with obesity or metabolically unhealthy status. Moreover, early management of metabolic health, along with targeted nutritional interventions, may serve as key strategies in mitigating the burden of UI in women affected by sarcopenia or sarcopenic obesity. Further longitudinal and prospective studies are needed to confirm these findings and elucidate the underlying mechanisms.

## Data Availability

Publicly available datasets were analyzed in this study. This data can be found: NHANES website (http://www.cdc.gov/nchs/nhanes).

## Footnotes

^1^ https://wwwn.cdc.gov/nchs/nhanes/tutorials/default.aspx
