# Supplementary material for: Associations of sarcopenia, obesity, and metabolic health with the risk of urinary incontinence in U.S. adult women: a population-based cross-sectional study
Source: Front Nutr. 2024 Oct 14;11:1459641. doi: 10.3389/fnut.2024.1459641 (PMC11513287; doi:10.3389/fnut.2024.1459641)
Supplement: Supplementary file 1 [file Data_Sheet_1.docx]

Supplementary Material

**Supplementary Figure 1.** **Density distribution of DXA parameters in 5 imputed datasets.**

**
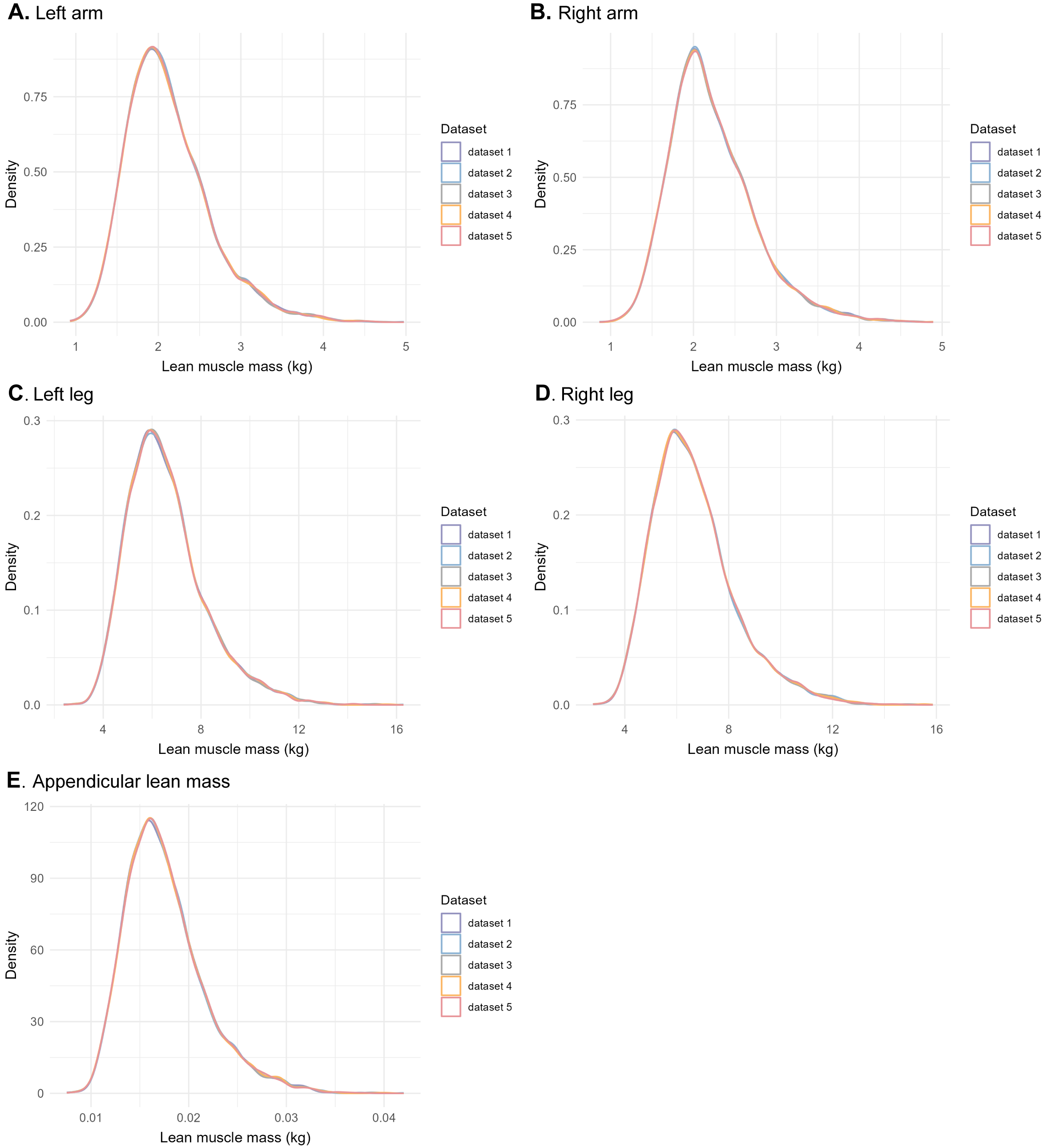
**

Density distribution of the lean muscle mass of the A. left arm, B. right arm, C. left leg, D. right leg, and E. total appendicular lean mass. The five colored curves correspond to five separate imputed datasets.

**Supplementary Figure 2: RCS analysis for associations between ALMI and UI subtypes across different age groups.**

**
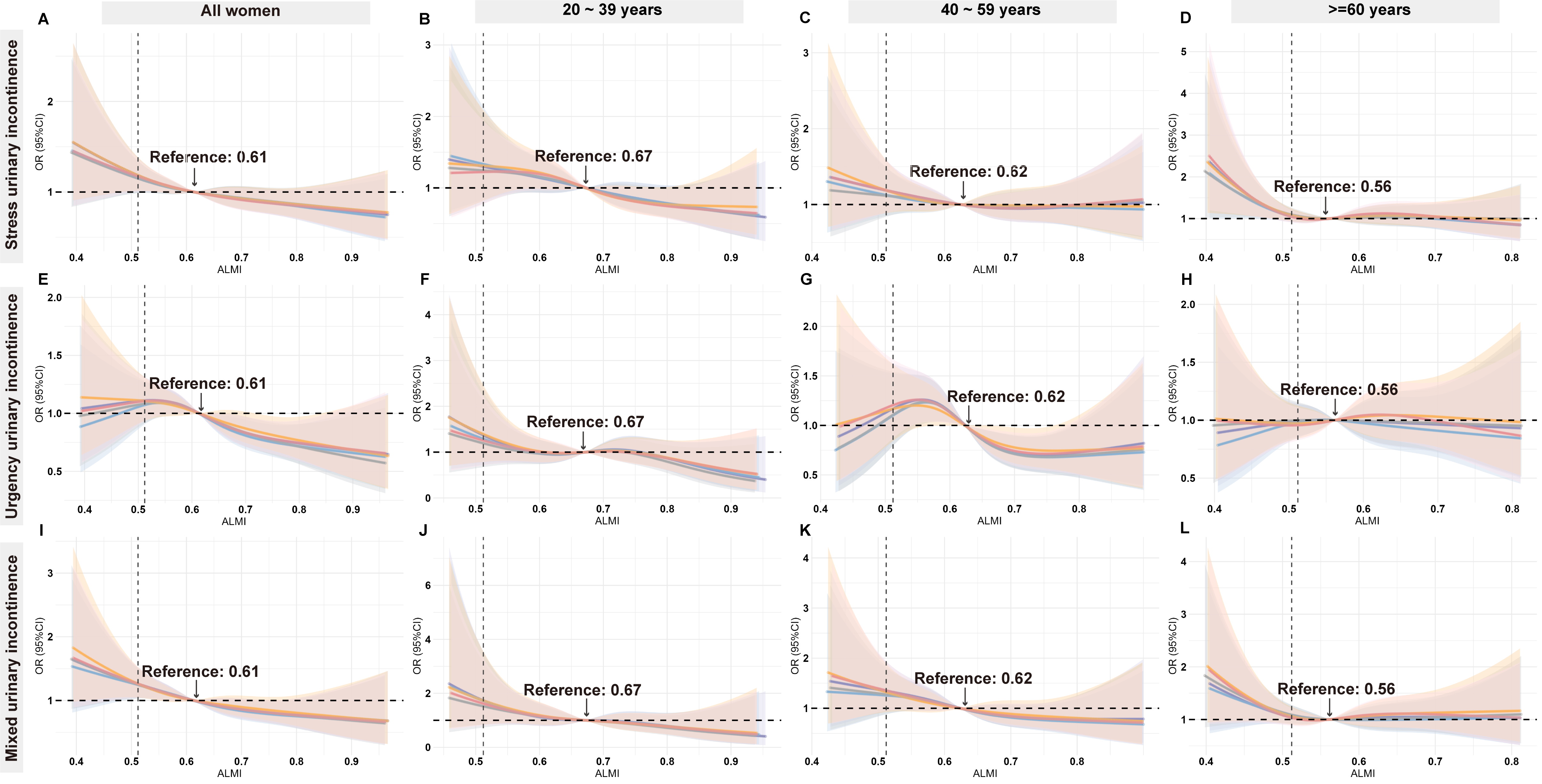
**

(A)-(D) correspond to stress urinary incontinence, (E)-(H) to urgency urinary incontinence, and (I)-(L) to mixed urinary incontinence in all women and then stratified by age groups: 20-39 years, 40-59 years, and 60 years and above. The Y-axis displays OR, while the X-axis represents ALMI values. The solid line and shaded areas indicate the estimated OR and the corresponding 95% CI, respectively. Each figure comprises 5 differently colored lines and shaded area, with each unique color representing one distinct imputed dataset. The data were fitted using a restricted cubic spline logistic model applied with 4 knots positioned at the 5th, 35th, 65th, and 95th percentiles of ALMI, with the reference ALMI set at OR=1. All models were adjusted for age (continuous), race/ethnicity, marital status, educational level, family poverty ratio, smoking behavior, physical activities, diabetes, parity, and female hormone use.

Abbreviations: ALMI, Appendicular lean mass index; OR, odds ratio; CI, confidence interval.

**Supplementary Table 1. Baseline characteristics of participants among different UI subtypes (weighted).**

| **Characteristics** | **Stress urinary incontinence** | | | **Urgency urinary incontinence** | | | **Mixed urinary incontinence** | | |
| --- | --- | --- | --- | --- | --- | --- | --- | --- | --- |
|  | **Non-SUI**  **(N=2,153)** | **SUI**  **(N=1,404)** | ***P*-**  **value** | **Non-UUI**  **(N=2,605)** | **UUI**  **(N=952)** | ***P*-**  **value** | **Non-MUI**  **(N=2,936)** | **MUI**  **(N=621)** | ***P*-**  **value** |
| **Age (year), mean (SD)** | 43.0 (16.9) | 50.1 (15.2) | **<0.001*** | 43.5 (16.0) | 53.2 (16.6) | **<0.001*** | 44.2 (16.2) | 54.5 (16.0) | **<0.001*** |
| **Age categories, N (%)** |  |  |  |  |  |  |  |  |  |
| 20 ~ 39 years | 874 (48.4) | 284 (24.7) | **<0.001*** | 1006 (44.4) | 152 (21.2) | **<0.001*** | 1075 (42.8) | 83 (17.9) | **<0.001*** |
| 40 ~ 59 years | 577 (32.9) | 578 (49.6) |  | 837 (38.4) | 318 (43.4) |  | 942 (38.7) | 213 (43.9) |  |
| ≥ 60 years | 702 (18.6) | 542 (25.7) |  | 762 (17.2) | 482 (35.4) |  | 919 (18.5) | 325 (38.1) |  |
| **Race/ethnicity, N (%)** |  |  |  |  |  |  |  |  |  |
| Mexican American | 427 (6.9) | 317 (6.8) | **<0.001*** | 540 (7.0) | 204 (6.2) | 0.810 | 607 (7.0) | 137 (6.0) | 0.152 |
| Non-Hispanic White | 1018 (67.8) | 804 (77.5) |  | 1319 (71.5) | 503 (72.1) |  | 1476 (70.9) | 346 (76.0) |  |
| Non-Hispanic Black | 527 (14.1) | 193 (7.8) |  | 540 (11.6) | 180 (11.9) |  | 620 (11.9) | 100 (10.3) |  |
| Others | 181 (11.2) | 90 (7.9) |  | 206 (9.9) | 65 (9.7) |  | 233 (10.3) | 38 (7.7) |  |
| **Marital status, N (%)** |  |  |  |  |  |  |  |  |  |
| Married / living with partner | 1141 (57.3) | 824 (65.8) | **<0.001*** | 1500 (62.4) | 465 (54.9) | **0.003*** | 1658 (61.5) | 307 (56.3) | **0.038*** |
| Separated / living alone | 1012 (42.7) | 580 (34.2) |  | 1105 (37.6) | 487 (45.1) |  | 1278 (38.5) | 314 (43.7) |  |
| **Educational level, N (%)** |  |  |  |  |  |  |  |  |  |
| Less than high school | 601 (16.7) | 412 (18.8) | 0.302 | 688 (16.0) | 325 (22.5) | **<0.001*** | 787 (16.2) | 226 (24.8) | **<0.001*** |
| High school | 517 (25.3) | 355 (25.8) |  | 629 (24.9) | 243 (27.4) |  | 715 (25.0) | 157 (28.2) |  |
| More than high school | 1035 (58.0) | 637 (55.4) |  | 1288 (59.0) | 384 (50.1) |  | 1434 (58.7) | 238 (47.1) |  |
| **Family PIR, N (%)** |  |  |  |  |  |  |  |  |  |
| < 1.3 | 581 (21.0) | 382 (20.7) | 0.068 | 664 (19.4) | 299 (25.9) | **0.018*** | 752 (20.0) | 211 (26.2) | **0.027*** |
| 1.3 ~ 3.5 | 824 (36.8) | 501 (33.4) |  | 971 (35.7) | 354 (34.7) |  | 1102 (35.5) | 223 (35.5) |  |
| > 3.5 | 607 (35.8) | 436 (40.9) |  | 810 (39.1) | 233 (33.6) |  | 897 (38.6) | 146 (33.1) |  |
| Not recorded | 141 (6.3) | 85 (5.0) |  | 160 (5.8) | 66 (5.9) |  | 185 (5.9) | 41 (5.2) |  |
| **Smoking behavior, N (%)** |  |  |  |  |  |  |  |  |  |
| Never smoked | 1359 (59.1) | 788 (55.1) | **0.004*** | 1605 (58.5) | 542 (54.5) | **0.004*** | 1808 (58.4) | 339 (52.4) | **0.003*** |
| Former smoker | 366 (17.5) | 346 (23.3) |  | 471 (18.5) | 241 (24.2) |  | 547 (18.9) | 165 (25.2) |  |
| Current smoker | 428 (23.3) | 270 (21.6) |  | 529 (23.0) | 169 (21.4) |  | 581 (22.7) | 117 (22.4) |  |
| **Physical activity, N (%)** |  |  |  |  |  |  |  |  |  |
| Inactive | 1112 (45.6) | 780 (50.1) | **0.021*** | 1335 (46.0) | 557 (52.1) | **0.008*** | 1517 (45.9) | 375 (55.7) | **<0.001*** |
| Active | 943 (49.3) | 575 (46.7) |  | 1166 (49.9) | 352 (43.1) |  | 1302 (49.9) | 216 (39.3) |  |
| Not recorded | 98 (5.1) | 49 (3.1) |  | 104 (4.2) | 43 (4.8) |  | 117 (4.2) | 30 (4.9) |  |
| **BMI (kg/m^2^), mean (SD)** | 27.2 (6.7) | 29.5 (7.3) | **<0.001*** | 27.7 (6.9) | 29.6 (7.2) | **<0.001*** | 27.7 (6.9) | 30.3 (7.5) | **<0.001*** |
| **BMI categories, N (%)** |  |  |  |  |  |  |  |  |  |
| < 30 kg/m^2^ | 1488 (72.2) | 805 (59.9) | **<0.001*** | 1752 (69.7) | 541 (59.3) | **<0.001*** | 1963 (69.5) | 330 (55.2) | **<0.001*** |
| ≥ 30 kg/m^2^ | 665 (27.8) | 599 (40.1) |  | 853 (30.3) | 411 (40.7) |  | 973 (30.5) | 291 (44.8) |  |
| **WC (cm), mean (SD)** | 90.7 (15.3) | 96.9 (15.9) | **<0.001*** | 91.9 (15.6) | 97.2 (15.8) | **<0.001*** | 92.1 (15.5) | 99.1 (16.0) | **<0.001*** |
| **WC categories, N (%)** |  |  |  |  |  |  |  |  |  |
| < 88 cm | 878 (48.7) | 365 (29.6) | **<0.001*** | 1014 (45.1) | 229 (28.1) | **<0.001*** | 1115 (44.4) | 128 (23.2) | **<0.001*** |
| ≥ 88 cm | 1275 (51.3) | 1039 (70.4) |  | 1591 (54.9) | 723 (71.9) |  | 1821 (55.6) | 493 (76.8) |  |
| **Diabetes, N (%)** |  |  |  |  |  |  |  |  |  |
| Normal | 1504 (77.6) | 851 (68.9) | **<0.001*** | 1831 (76.9) | 524 (64.8) | **<0.001*** | 2028 (76.4) | 327 (61.6) | **<0.001*** |
| Prediabetes | 416 (15.1) | 337 (20.8) |  | 512 (16.0) | 241 (22.1) |  | 594 (16.3) | 159 (23.6) |  |
| Diabetes | 233 (7.3) | 216 (10.3) |  | 262 (7.1) | 187 (13.1) |  | 314 (7.3) | 135 (14.8) |  |
| **Hypertension, N (%)** |  |  |  |  |  |  |  |  |  |
| No | 1834 (90.3) | 1155 (85.2) | **<0.001*** | 2252 (90.0) | 737 (82.6) | **<0.001*** | 2511 (89.6) | 478 (80.7) | **<0.001*** |
| Yes | 319 (9.7) | 249 (14.8) |  | 353 (10.0) | 215 (17.4) |  | 425 (10.4) | 143 (19.3) |  |
| **Parity, N (%)** |  |  |  |  |  |  |  |  |  |
| 0 | 508 (29.5) | 163 (13.4) | **<0.001*** | 557 (25.6) | 114 (14.7) | **<0.001*** | 610 (25.1) | 61 (11.7) | **<0.001*** |
| 1 | 317 (16.1) | 182 (14.8) |  | 375 (15.8) | 124 (14.6) |  | 431 (16.2) | 68 (12.1) |  |
| 2 | 510 (26.5) | 382 (31.9) |  | 680 (29.3) | 212 (26.3) |  | 753 (28.8) | 139 (27.6) |  |
| 3 | 341 (14.9) | 311 (22.2) |  | 451 (16.7) | 201 (21.6) |  | 514 (16.9) | 138 (22.8) |  |
| ≥ 4 | 438 (12.1) | 348 (17.0) |  | 502 (11.7) | 284 (21.8) |  | 583 (12.2) | 203 (24.6) |  |
| Not recorded | 39 (0.9) | 18 (0.7) |  | 40 (0.8) | 17 (0.9) |  | 45 (0.8) | 12 (1.1) |  |
| **Female hormone use, N (%)** |  |  |  |  |  |  |  |  |  |
| No | 1639 (78.7) | 911 (64.8) | **<0.001*** | 1954 (76.7) | 596 (61.7) | **<0.001*** | 2176 (75.8) | 374 (58.3) | **<0.001*** |
| Yes | 469 (20.3) | 471 (34.1) |  | 606 (22.4) | 334 (37.0) |  | 708 (23.2) | 232 (40.1) |  |
| Not recorded | 45 (1.0) | 22 (1.1) |  | 45 (1.0) | 22 (1.3) |  | 52 (0.9) | 15 (1.7) |  |

Abbreviations: UI, urinary incontinence; SUI, stress urinary incontinence; UUI, urgency urinary incontinence; MUI, mixed urinary incontinence; BMI, body mass index; WC, waist circumference.

**P-*value < 0.05 is considered statistically significant and presented in bold text.

**Supplementary Table 2. Subgroup analysis of the association between sarcopenia and UI subtypes in all women (combined 5 imputed datasets).**

| **Subgroups** | **SUI**  **OR (95%CI)** | **UUI**  **OR (95%CI)** | **MUI**  **OR (95%CI)** |
| --- | --- | --- | --- |
| **Physical activity ^a^** |  |  |  |
| Inactive | **1.373 (1.006, 1.872) *** | 1.225 (0.790, 1.898) | **1.623 (1.087, 2.425) *** |
| Active | 1.010 (0.554, 1.840) | 1.138 (0.664, 1.948) | 1.470 (0.786, 2.750) |
| **Smoking behavior ^b^** |  |  |  |
| Never smoked | 1.187 (0.854, 1.648) | 0.948 (0.668, 1.346) | 1.328 (0.862, 2.046) |
| Former smoker | 1.462 (0.957, 2.233) | 1.611 (0.843, 3.078) | **2.282 (1.192, 4.366) *** |
| Current smoker | 1.340 (0.615, 2.917) | 1.654 (0.602, 4.546) | 1.781 (0.585, 5.418) |
| **Hypertension ^c^** |  |  |  |
| No | 1.338 (0.992, 1.805) | 1.457 (0.964, 2.202) | **1.915 (1.228, 2.987) *** |
| Yes | 1.114 (0.617, 2.012) | 0.672 (0.352, 1.285) | 0.899 (0.469, 1.722) |
| **Diabetes ^d^** |  |  |  |
| Normal | 0.894 (0.635, 1.259) | 1.147 (0.645, 2.040) | 1.253 (0.658, 2.386) |
| Prediabetes | **1.794 (1.069, 3.009) *** | 1.131 (0.727, 1.759) | **1.841 (1.085, 3.123) *** |
| Diabetes | 1.560 (0.800, 3.043) | 1.422 (0.715, 2.827) | **2.053 (1.057, 3.984) *** |
| **Parity ^e^** |  |  |  |
| 0 | 2.064 (0.804, 5.297) | 0.771 (0.286, 2.083) | 1.914 (0.651, 5.622) |
| 1 | 1.384 (0.661, 2.897) | 0.971 (0.424, 2.225) | 1.960 (0.762, 5.043) |
| 2 | **1.893 (1.142, 3.138) *** | 1.406 (0.679, 2.909) | 2.004 (0.919, 4.374) |
| 3 | 0.661 (0.356, 1.225) | 2.067 (0.891, 4.794) | **2.145 (1.012, 4.548) *** |
| ≥4 | 1.331 (0.857, 2.065) | 1.141 (0.694, 1.875) | 1.312 (0.739, 2.327) |

Abbreviations: UI, urinary incontinence; SUI, stress urinary incontinence; UUI, urgency urinary incontinence; MUI, mixed urinary incontinence; OR, odds ratio; CI, confidence interval.

^a^ Adjusted for age categories, race/ethnicity, marital status, educational level, family PIR, smoking behavior, diabetes, hypertension, parity, and female hormone use.

^b^ Adjusted for age categories, race/ethnicity, marital status, educational level, family PIR, physical activities, diabetes, hypertension, parity, and female hormone use.

^c^ Adjusted for age categories, race/ethnicity, marital status, educational level, family PIR, physical activities, smoking behavior, diabetes, parity, and female hormone use.

^d^ Adjusted for age categories, race/ethnicity, marital status, educational level, family PIR, physical activities, smoking behavior, hypertension, parity, and female hormone use.

^e^ Adjusted for age categories, race/ethnicity, marital status, educational level, family PIR, physical activities, smoking behavior, hypertension, diabetes, and female hormone use.

**Supplementary Table 3. The mediation analysis of metabolic indicators on the association between sarcopenia and mixed urinary incontinence (imputed dataset 2-5).**

| **Mediators** | **Direct effect**  **β (95% CI)** | ***P*-value** | **Indirect effect**  **β (95% CI)** | ***P*-value** | **Mediated proportion**  **% (95%CI)** | ***P*-value** |
| --- | --- | --- | --- | --- | --- | --- |
| **Metabolically unhealthy status ^a^** |  |  |  |  |  |  |
| Dataset 2 | 0.059 (0.020, 0.098) | **0.004*** | 0.003 (0.000, 0.006) | **0.026*** | 4.21% (0.23%, 16.43%) | **0.026*** |
| Dataset 3 | 0.066 (0.028, 0.107) | **<0.001*** | 0.003 (0.000, 0.006) | **0.028*** | 3.74% (0.14%, 12.68%) | **0.028*** |
| Dataset 4 | 0.065 (0.026, 0.105) | **<0.001*** | 0.003 (0.000, 0.006) | **0.026*** | 3.73% (0.23%, 12.35%) | **0.026*** |
| Dataset 5 | 0.058 (0.020, 0.098) | **0.002*** | 0.003 (0.000, 0.006) | **0.026*** | 4.36% (0.21%, 14.85%) | **0.028*** |
| **Hemoglobin A1c (HbA1c) ^b^** |  |  |  |  |  |  |
| Dataset 2 | 0.060 (0.020, 0.101) | **0.004*** | 0.004 (0.001, 0.007) | **0.002*** | 6.56% (1.97%, 19.79%) | **0.002*** |
| Dataset 3 | 0.067 (0.025, 0.109) | **<0.001*** | 0.004 (0.001, 0.007) | **<0.001*** | 5.37% (1.36%, 13.97%) | **<0.001*** |
| Dataset 4 | 0.063 (0.023, 0.107) | **<0.001*** | 0.004 (0.001, 0.008) | **<0.001*** | 6.09% (1.57%, 17.46%) | **<0.001*** |
| Dataset 5 | 0.056 (0.018, 0.099) | **<0.001*** | 0.004 (0.001, 0.007) | **0.002*** | 6.58% (1.71%, 19.91%) | **0.002*** |
| **Vitamin D ^c^** |  |  |  |  |  |  |
| Dataset 2 | 0.052 (0.013, 0.093) | **0.008*** | 0.003 (0.001, 0.007) | **0.008*** | 5.72% (1.05%, 19.45%) | **0.014*** |
| Dataset 3 | 0.060 (0.021, 0.102) | **0.004*** | 0.003 (0.001, 0.006) | **0.010*** | 5.02% (0.96%, 15.31%) | **0.012*** |
| Dataset 4 | 0.057 (0.017, 0.098) | **0.006*** | 0.003 (0.000, 0.006) | **0.014*** | 4.55% (0.55%, 16.08%) | **0.018*** |
| Dataset 5 | 0.050 (0.010, 0.089) | **0.016*** | 0.004 (0.001, 0.007) | **0.004*** | 6.68% (1.35%, 25.71%) | **0.014*** |
| **Albmin ^d^** |  |  |  |  |  |  |
| Dataset 2 | 0.048 (0.010, 0.088) | **0.020*** | 0.006 (0.002, 0.010) | **<0.001*** | 10.33% (3.03%, 35.74%) | **0.006*** |
| Dataset 3 | 0.057 (0.017, 0.098) | **<0.001*** | 0.005 (0.001, 0.009) | **<0.001*** | 7.75% (2.25%, 23.16%) | **<0.001*** |
| Dataset 4 | 0.055 (0.015, 0.097) | **0.004*** | 0.006 (0.002, 0.010) | **<0.001*** | 9.32% (2.73%, 29.31%) | **0.004*** |
| Dataset 5 | 0.048 (0.009, 0.088) | **0.006*** | 0.005 (0.001, 0.010) | **<0.001*** | 9.55% (2.77%, 38.36%) | **0.002*** |

^a^ This mediation analysis using sample size: 3327. The model was adjusted for age categories, race/ethnicity, marital status, educational level, family PIR, smoking behavior, physical activities, parity, and female hormone use.

^b^ This mediation analysis using sample size: 3389. The model was adjusted for age categories, race/ethnicity, marital status, educational level, family PIR, smoking behavior, physical activities, hypertension, parity, and female hormone use.

^c^ This mediation analysis using sample size: 3331. The model was adjusted for age categories, race/ethnicity, marital status, educational level, family PIR, smoking behavior, physical activities, hypertension, diabetes, parity, and female hormone use.

^d^ This mediation analysis using sample size: 3325. The model was adjusted for age categories, race/ethnicity, marital status, educational level, family PIR, smoking behavior, physical activities, hypertension, diabetes, parity, and female hormone use.

**Supplementary Table 4. The mediation analysis of metabolic indicators on the association between sarcopenic obesity and stress urinary incontinence (imputed dataset 2-5).**

| **Mediators** | **Direct effect**  **β (95% CI)** | ***P*-**  **value** | **Indirect effect**  **β (95% CI)** | ***P*-**  **value** | **Mediated proportion**  **% (95%CI)** | ***P*-**  **value** |
| --- | --- | --- | --- | --- | --- | --- |
| **Sarcopenic obesity (obesity defined by BMI)** | | | | | | |
| **Metabolically unhealthy status ^a^** |  |  |  |  |  |  |
| Dataset 2 | 0.041 (0.023, 0.058) | **<0.001*** | 0.005 (0.002, 0.009) | **0.002*** | 11.44% (4.24%, 22.29%) | **0.002*** |
| Dataset 3 | 0.044 (0.027, 0.062) | **<0.001*** | 0.005 (0.002, 0.009) | **0.002*** | 10.29% (3.98%, 20.05%) | **0.002*** |
| Dataset 4 | 0.042 (0.024, 0.060) | **<0.001*** | 0.005 (0.002, 0.009) | **<0.001*** | 11.20% (4.19%, 21.22%) | **<0.001*** |
| Dataset 5 | 0.042 (0.024, 0.060) | **<0.001*** | 0.005 (0.002, 0.009) | **0.002*** | 10.88% (4.35%, 22.00%) | **0.002*** |
| **Hemoglobin A1c (HbA1c) ^b^** |  |  |  |  |  |  |
| Dataset 2 | 0.046 (0.028, 0.061) | **<0.001*** | 0.004 (0.001, 0.007) | **<0.001*** | 7.65% (2.37%, 16.47%) | **<0.001*** |
| Dataset 3 | 0.048 (0.031, 0.064) | **<0.001*** | 0.004 (0.001, 0.006) | **<0.001*** | 7.00% (2.23%, 14.55%) | **<0.001*** |
| Dataset 4 | 0.046 (0.029, 0.062) | **<0.001*** | 0.004 (0.001, 0.007) | **<0.001*** | 7.59% (2.32%, 15.88%) | **<0.001*** |
| Dataset 5 | 0.046 (0.029, 0.062) | **<0.001*** | 0.004 (0.001, 0.007) | **<0.001*** | 7.39% (2.23%, 15.39%) | **<0.001*** |
| **Vitamin D ^c^** |  |  |  |  |  |  |
| Dataset 2 | 0.040 (0.022, 0.056) | **<0.001*** | 0.003 (0.000, 0.006) | **0.044*** | 6.42% (0.20%, 15.73%) | **0.044*** |
| Dataset 3 | 0.043 (0.025, 0.058) | **<0.001*** | 0.003 (0.000, 0.005) | 0.050 | 5.87% (0.10%, 13.94%) | 0.05 |
| Dataset 4 | 0.041 (0.023, 0.057) | **<0.001*** | 0.003 (0.000, 0.005) | **0.046*** | 6.18% (0.28%, 15.21%) | **0.046*** |
| Dataset 5 | 0.042(0.023, 0.057) | **<0.001*** | 0.003 (0.000, 0.006) | 0.050 | 6.22% (0.09%, 15.15%) | 0.050 |
| **Albumin ^d^** |  |  |  |  |  |  |
| Dataset 2 | 0.039 (0.020, 0.058) | **<0.001*** | 0.003 (-0.000, 0.007) | 0.086 | 7.53% (-0.92%, 20.88%) | 0.086 |
| Dataset 3 | 0.042 (0.023, 0.061) | **<0.001*** | 0.003 (-0.000, 0.007) | 0.088 | 6.62% (-0.80%, 17.88%) | 0.088 |
| Dataset 4 | 0.040 (0.021, 0.059) | **<0.001*** | 0.003 (-0.001, 0.007) | 0.084 | 7.32% (-1.04%, 19.82%) | 0.084 |
| Dataset 5 | 0.041 (0.022, 0.059) | **<0.001*** | 0.003 (-0.000, 0.007) | 0.084 | 6.97% (-0.82%, 18.72%) | 0.084 |
| **Sarcopenic obesity (obesity defined by WC)** | | | | | | |
| **Metabolically unhealthy status ^a^** |  |  |  |  |  |  |
| Dataset 2 | 0.043 (0.026, 0.061) | **<0.001*** | 0.006 (0.002, 0.009) | **0.002*** | 11.35% (4.08%, 22.26%) | **0.002*** |
| Dataset 3 | 0.046 (0.030, 0.063) | **<0.001*** | 0.005 (0.002, 0.009) | **0.002*** | 10.33% (3.69%, 19.90%) | **0.002*** |
| Dataset 4 | 0.044 (0.028, 0.062) | **<0.001*** | 0.005 (0.002, 0.009) | **0.002*** | 10.85% (4.09%, 21.56%) | **0.002*** |
| Dataset 5 | 0.045 (0.027, 0.062) | **<0.001*** | 0.005 (0.002, 0.009) | **0.002*** | 10.98% (4.07%, 21.58%) | **0.002*** |
| **Hemoglobin A1c (HbA1c) ^b^** |  |  |  |  |  |  |
| Dataset 2 | 0.048 (0.030, 0.063) | **<0.001*** | 0.004 (0.001, 0.006) | **<0.001*** | 7.01% (2.46%, 14.50%) | **<0.001*** |
| Dataset 3 | 0.050 (0.034, 0.065) | **<0.001*** | 0.003 (0.001, 0.006) | **0.002*** | 6.40% (2.21%, 12.91%) | **0.002*** |
| Dataset 4 | 0.049 (0.031, 0.064) | **<0.001*** | 0.004 (0.001, 0.006) | **<0.001*** | 6.94% (2.38%, 14.45%) | **<0.001*** |
| Dataset 5 | 0.049 (0.032, 0.064) | **<0.001*** | 0.004 (0.001, 0.006) | **<0.001*** | 6.76% (2.37%, 13.90%) | **<0.001*** |
| **Vitamin D ^c^** |  |  |  |  |  |  |
| Dataset 2 | 0.044 (0.027, 0.060) | **<0.001*** | 0.003 (0.000, 0.005) | **0.048*** | 5.51% (0.13%, 13.50%) | **0.048*** |
| Dataset 3 | 0.047 (0.029, 0.063) | **<0.001*** | 0.003 (0.000, 0.005) | 0.050 | 5.06% (0.05%, 12.01%) | 0.050 |
| Dataset 4 | 0.045 (0.028, 0.061) | **<0.001*** | 0.003 (0.000, 0.005) | **0.046*** | 5.28% (0.12%, 12.64%) | **0.046*** |
| Dataset 5 | 0.046 (0.028, 0.062) | **<0.001*** | 0.003 (0.000, 0.005) | **0.048*** | 5.36% (0.06%, 12.63%) | **0.048*** |
| **Albumin ^d^** |  |  |  |  |  |  |
| Dataset 2 | 0.041 (0.024, 0.059) | **<0.001*** | 0.003 (-0.000, 0.007) | 0.068 | 6.69% (-0.67%, 17.21%) | 0.068 |
| Dataset 3 | 0.044 (0.026, 0.061) | **<0.001*** | 0.003 (-0.000, 0.006) | 0.072 | 5.87% (-0.66%, 14.57%) | 0.072 |
| Dataset 4 | 0.043 (0.025, 0.060) | **<0.001*** | 0.003 (-0.000, 0.006) | 0.072 | 6.49% (-0.84%, 15.98%) | 0.072 |
| Dataset 5 | 0.043 (0.026, 0.060) | **<0.001*** | 0.003 (-0.000, 0.006) | 0.070 | 6.18% (-0.65%, 15.77%) | 0.070 |

^a^ This mediation analysis using sample size: 3327. The model was adjusted for age categories, race/ethnicity, marital status, educational level, family PIR, smoking behavior, physical activities, parity, and female hormone use.

^b^ This mediation analysis using sample size: 3389. The model was adjusted for age categories, race/ethnicity, marital status, educational level, family PIR, smoking behavior, physical activities, hypertension, parity, and female hormone use.

^c^ This mediation analysis using sample size: 3331. The model was adjusted for age categories, race/ethnicity, marital status, educational level, family PIR, smoking behavior, physical activities, hypertension, diabetes, parity, and female hormone use.

^d^ This mediation analysis using sample size: 3325. The model was adjusted for age categories, race/ethnicity, marital status, educational level, family PIR, smoking behavior, physical activities, hypertension, diabetes, parity, and female hormone use.

**Supplementary Table 5. The mediation analysis of metabolic indicators on the association between sarcopenic obesity and mixed urinary incontinence (imputed dataset 2 - 5).**

| **Mediators** | **Direct effect**  **β (95% CI)** | ***P*-**  **value** | **Indirect effect**  **β (95% CI)** | ***P*-**  **value** | **Mediated proportion**  **% (95%CI)** | ***P*-**  **value** |
| --- | --- | --- | --- | --- | --- | --- |
| **Sarcopenic obesity (obesity defined by BMI)** | | | | | | |
| **Metabolically unhealthy status ^a^** |  |  |  |  |  |  |
| Dataset 2 | 0.030 (0.020, 0.039) | **<0.001*** | 0.001 (-0.001, 0.004) | 0.242 | 3.79% (-2.21%, 12.30%) | 0.242 |
| Dataset 3 | 0.032 (0.022, 0.040) | **<0.001*** | 0.001 (-0.001, 0.004) | 0.256 | 3.40% (-2.12%, 11.60%) | 0.256 |
| Dataset 4 | 0.031 (0.021, 0.040) | **<0.001*** | 0.001 (-0.001, 0.003) | 0.240 | 3.58% (-1.93%, 11.42%) | 0.240 |
| Dataset 5 | 0.030 (0.020, 0.039) | **<0.001*** | 0.001 (-0.001, 0.004) | 0.230 | 3.80% (-2.10%, 12.31%) | 0.230 |
| **Hemoglobin A1c (HbA1c) ^b^** |  |  |  |  |  |  |
| Dataset 2 | 0.031 (0.021, 0.039) | **<0.001*** | 0.002 (0.001, 0.004) | **<0.001*** | 6.25% (1.87%, 12.26%) | **<0.001*** |
| Dataset 3 | 0.032 (0.022, 0.040) | **<0.001*** | 0.002 (0.001, 0.003) | **0.002*** | 5.76% (1.76%, 11.27%) | **0.002*** |
| Dataset 4 | 0.031 (0.022, 0.039) | **<0.001*** | 0.002 (0.001, 0.004) | **0.002*** | 6.08% (1.88%, 12.17%) | **0.002*** |
| Dataset 5 | 0.030 (0.020, 0.038) | **<0.001*** | 0.002 (0.001, 0.004) | **<0.001*** | 6.34% (2.04%, 12.84%) | **<0.001*** |
| **Vitamin D ^c^** |  |  |  |  |  |  |
| Dataset 2 | 0.027 (0.017, 0.036) | **<0.001*** | 0.002 (0.000, 0.004) | **0.008*** | 7.99% (1.51%, 17.21%) | **0.008*** |
| Dataset 3 | 0.029 (0.018, 0.037) | **<0.001*** | 0.002 (0.000, 0.004) | **0.010*** | 7.36% (1.22%, 16.09%) | **0.010*** |
| Dataset 4 | 0.028 (0.018, 0.037) | **<0.001*** | 0.002 (0.000, 0.004) | **0.008*** | 7.47% (1.33%, 16.36%) | **0.008*** |
| Dataset 5 | 0.027 (0.015, 0.036) | **<0.001*** | 0.002 (0.000, 0.005) | **0.008*** | 8.33% (1.53%, 17.98%) | **0.008*** |
| **Albumin ^d^** |  |  |  |  |  |  |
| Dataset 2 | 0.026 (0.015, 0.036) | **<0.001*** | 0.003 (0.001, 0.006) | **0.008*** | 10.99% (2.73%, 23.53%) | **0.008*** |
| Dataset 3 | 0.028 (0.017, 0.037) | **<0.001*** | 0.003 (0.001, 0.005) | **0.008*** | 9.80% (2.18%, 20.70%) | **0.008*** |
| Dataset 4 | 0.027 (0.016, 0.037) | **<0.001*** | 0.003 (0.001, 0.006) | **0.008*** | 10.36% (2.26%, 22.06%) | **0.008*** |
| Dataset 5 | 0.026 (0.015, 0.035) | **<0.001*** | 0.003 (0.001, 0.006) | **0.008*** | 10.78% (2.73%, 23.48%) | **0.008*** |
| **Sarcopenic obesity (obesity defined by WC)** | | | | | | |
| **Metabolically unhealthy status ^a^** |  |  |  |  |  |  |
| Dataset 2 | 0.030 (0.021, 0.038) | **<0.001*** | 0.001 (-0.001, 0.003) | 0.290 | 3.59% (-2.72%, 11.87%) | 0.290 |
| Dataset 3 | 0.031 (0.023, 0.038) | **<0.001*** | 0.001 (-0.001, 0.003) | 0.322 | 3.22% (-2.74%, 11.21%) | 0.322 |
| Dataset 4 | 0.031 (0.022, 0.038) | **<0.001*** | 0.001 (-0.001, 0.003) | 0.308 | 3.26% (-2.66%, 11.80%) | 0.308 |
| Dataset 5 | 0.030 (0.021, 0.037) | **<0.001*** | 0.001 (-0.001, 0.003) | 0.288 | 3.67% (-2.71%, 12.57%) | 0.288 |
| **Hemoglobin A1c (HbA1c) ^b^** |  |  |  |  |  |  |
| Dataset 2 | 0.030 (0.021, 0.037) | **<0.001*** | 0.002 (0.001, 0.003) | **<0.001*** | 6.02% (2.00%, 12.45%) | **<0.001*** |
| Dataset 3 | 0.031 (0.022, 0.038) | **<0.001*** | 0.002 (0.001, 0.003) | **<0.001*** | 5.51% (1.85%, 11.15%) | **<0.001*** |
| Dataset 4 | 0.031 (0.021, 0.038) | **<0.001*** | 0.002 (0.001, 0.003) | **<0.001*** | 5.82% (1.93%, 11.75%) | **<0.001*** |
| Dataset 5 | 0.030 (0.020, 0.037) | **<0.001*** | 0.002 (0.001, 0.003) | **<0.001*** | 6.08% (2.10%, 12.48%) | **<0.001*** |
| **Vitamin D ^c^** |  |  |  |  |  |  |
| Dataset 2 | 0.028 (0.018, 0.035) | **<0.001*** | 0.002 (0.000, 0.004) | **0.002*** | 7.30% (1.75%, 16.47%) | **0.002*** |
| Dataset 3 | 0.029 (0.020, 0.037) | **<0.001*** | 0.002 (0.000, 0.004) | **0.006*** | 6.74% (1.43%, 14.97%) | **0.006*** |
| Dataset 4 | 0.029 (0.019, 0.036) | **<0.001*** | 0.002 (0.000, 0.004) | **0.002*** | 6.77% (1.47%, 14.61%) | **0.002*** |
| Dataset 5 | 0.027 (0.017, 0.035) | **<0.001*** | 0.002 (0.000, 0.004) | **0.004*** | 7.61% (1.61%, 17.19%) | **0.004*** |
| **Albumin ^d^** |  |  |  |  |  |  |
| Dataset 2 | 0.027 (0.016, 0.035) | **<0.001*** | 0.003 (0.001, 0.005) | **0.004*** | 9.92% (2.37%, 20.92%) | **0.004*** |
| Dataset 3 | 0.028 (0.018, 0.036) | **<0.001*** | 0.003 (0.001, 0.005) | **0.004*** | 8.79% (1.93%, 17.96%) | **0.004*** |
| Dataset 4 | 0.028 (0.017, 0.036) | **<0.001*** | 0.003 (0.001, 0.005) | **0.004*** | 9.33% (2.13%, 19.26%) | **0.004*** |
| Dataset 5 | 0.026 (0.016, 0.034) | **<0.001*** | 0.003 (0.001, 0.005) | **0.004*** | 9.67% (2.31%, 20.25%) | **0.004*** |

^a^ This mediation analysis using sample size: 3327. The model was adjusted for age categories, race/ethnicity, marital status, educational level, family PIR, smoking behavior, physical activities, parity, and female hormone use.

^b^ This mediation analysis using sample size: 3389. The model was adjusted for age categories, race/ethnicity, marital status, educational level, family PIR, smoking behavior, physical activities, hypertension, parity, and female hormone use.

^c^ This mediation analysis using sample size: 3331. The model was adjusted for age categories, race/ethnicity, marital status, educational level, family PIR, smoking behavior, physical activities, hypertension, diabetes, parity, history of hysterectomy, and female hormone use.

^d^ This mediation analysis using sample size: 3325. The model was adjusted for age categories, race/ethnicity, marital status, educational level, family PIR, smoking behavior, physical activities, hypertension, diabetes, parity, history of hysterectomy, and female hormone use.
